# Multiple ETS family transcription factors bind mutant p53 *via* distinct interaction regions

**DOI:** 10.1002/1873-3468.70260

**Published:** 2025-12-31

**Authors:** Stephanie A. Metcalf, Nicholas F. Downing, Kaitlyn M. Mills, Samuel C. Metcalfe, Alexander E. Kritzer, Lindsey D. Mayo, Peter C. Hollenhorst

**Affiliations:** ^1^ Medical Sciences Indiana University School of Medicine Bloomington IN USA; ^2^ Department of Pediatrics Indiana University School of Medicine Indianapolis IN USA

**Keywords:** ETS family, mutant p53, ovarian cancer, prostate cancer

## Abstract

ETS family transcription factors can mediate mutant p53 functions, but there has been no comprehensive analysis of p53 interaction across the ETS family. By comparing direct mutant p53 binding between 26 ETS proteins, we found that all bound mutant p53, but relative binding differed significantly. The ETS DNA binding domain provided a common interaction interface, but strong binding required an alternate interaction domain highlighted by a PXXPP motif found in five ETS proteins. Genome‐wide mapping found that the ETS protein ERG mediated some mutant p53 DNA binding in prostate cancer cells. Lastly, ETS proteins that interact strongly with mutant p53 tended to be upregulated in p53 mutant ovarian cancer. These results identify multiple ETS family members that could mediate mutant p53 function in cancer.

**Impact statement**

The mechanisms behind gain‐of‐function mutant p53 remain unclear. Here we identify distinct domains and a novel motif that can mediate binding of mutant p53 to multiple different ETS family transcription factors.

## Graphical abstract

Mutant p53 gain‐of‐function is thought to be mediated by interaction with other transcription factors. We identify multiple ETS transcription factors that can bind mutant p53 and found that this interaction can be promoted by a PXXPP motif. ETS proteins that strongly bound mutant p53 were upregulated in ovarian cancer compared to ETS proteins that bound mutant p53 weakly.

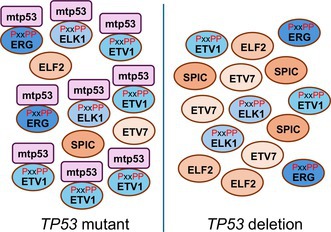

## Abbreviations

**DTT**, dithiothreitol

**HGSOC**, high‐grade serous ovarian cancer

**wt**, wild‐type

The *TP53* gene is one of the most altered in cancer, with mutations or deletions occurring in approximately half of all cancer cases [1]. *TP53* encodes p53, a transcription factor with tumor suppressive functions [2]. The tumor suppressive activity can be lost through gene deletion or missense mutation [3]. However, mutations in the *TP53* gene are more common than deletion. Some missense mutations in *TP53* are associated with more aggressive cancer phenotypes and poorer prognosis than *TP53* deletion [1, 3, 4, 5]. Further, studies that delete mutant p53 in mouse models have found reduced cancer‐associated phenotypes including decreased tumor growth [6, 7, 8, 9]. This suggests that mutant p53 has a gain of oncogenic function in addition to the loss of the tumor suppressive role of wild‐type (wt) p53 [1, 3, 4]. Any gain of function role for mutant p53 appears to be cell/tumor‐specific, as some studies find that deletion of mutant p53 has no effect [10]. The specific contexts where mutant p53 has oncogenic roles still need to be determined.

In male and female cancers of the reproductive organs, *TP53* is mutated frequently. Specifically, in high‐grade serous ovarian cancer (HGSOC), the most common and deadly subtype, *TP53* disruption occurs in nearly 100% of cases [11]. In other subtypes of ovarian cancer, *TP53* is the most mutated gene, with alterations in about 70% of cases. In ovarian cancer, gain‐of‐function p53 is associated with therapy resistance [12], and specific p53 mutations, such as R248W, are associated with poor overall survival [13]. In metastatic castration‐resistant prostate cancer, the *TP53* mutation rate is over 50%, and these mutations contribute to therapy resistance [14, 15]. In breast cancer, mutant p53 promotes disruption of tissue architecture [6] and deletion of mutant p53 can decrease tumor growth [9].

Missense p53 mutations in the DNA binding domain reduce or eliminate direct DNA binding to consensus sequences in target genes responsible for regulating numerous cellular outcomes [16]. However, mutant p53 can regulate new sets of target genes by binding the genome *via* interaction with other transcription factors [17, 18]. Mapping mutant p53 genomic occupancy identified enrichment for ETS transcription factor binding sites [19, 20]. The ETS family transcription factor ETS2 can interact with mutant p53 resulting in gain‐of‐function p53 activity [19]. ETS2 can directly bind mutant p53 (R248W and R175H) better than wt p53 [19]. Other ETS family transcription factors, ETS1, ERG, and ELK1 can bind mutant p53 in co‐immunoprecipitation experiments [19, 21, 22], but tests of direct protein–protein interaction have not been reported. ETS2 is important for mutant p53 mediated activation of PLA2G16 phospholipase and small nucleolar RNAs (snoRNAs) in osteosarcoma cells [23, 24]. ELK1 is important for mutant p53 mediated activation of FRA‐1 in breast cancer [22]. ERG can cooperate with mutant p53 to drive prostate adenocarcinoma [25]. Therefore, multiple ETS transcription factors have the potential to recruit mutant p53.

The ETS transcription factor family is comprised of 28 members in humans that have a conserved ETS DNA binding domain and bind to a similar consensus DNA sequence [26]. ETS proteins are responsible for cell cycle control, differentiation, proliferation, apoptosis, tissue remodeling, and angiogenesis [26]. Multiple ETS family members can promote cancer, including ETS2, ERG, and ETV1. ETS2 is ubiquitously expressed and can have both oncogenic and tumor suppressive roles depending on cellular context [27]. Aberrant expression of ERG, driven by the *TMPRSS2/ERG* gene fusion, occurs in one‐half of prostate tumors and is a disease driver [21]. ERG‐positive prostate cancers are correlated with p53 alterations [21]. An additional ~ 10% of prostate tumors have gene fusions that result in aberrant expression of ETV1 [28]. Like ERG, ETV1 expression in prostate cells is oncogenic [28, 29, 30]. ETV1 is also overexpressed in triple‐negative breast cancer, and this overexpression is important for cellular migration and proliferation [31]. Incidentally, a large portion (60%) of triple‐negative breast cancer patients harbor a p53 mutation which is also associated with poorer outcomes [32]. Therefore, several oncogenic ETS proteins play roles in cancer types that also have a high incidence of p53 mutations.

The ability of mutant p53 to interact with multiple members of the ETS family of transcription factors raises the possibility that specific ETS/mutant p53 complexes target specific genes. This study examined direct protein–protein interactions between 26 of the 28 ETS transcription factors and three p53 gain‐of‐function mutants. All ETS proteins interacted with mutant p53, and a subset interacted more strongly than ETS2. The ETS DNA binding domain allowed common interaction, but strong binding required a second, alternate interaction domain in the ETS protein. In five ETS proteins, this second domain contained a critical PXXPP motif. Genome‐wide mapping identified a role for ERG in the recruitment of mutant p53 to specific genomic regions in a prostate cancer cell line. Strikingly, we found that there is a correlation between *TP53* mutation and upregulation of the ETS proteins that we identified as strong mutant p53 interactors in ovarian cancer. Taken together, these findings suggest a dynamic interplay between multiple ETS proteins and mutant p53 *via* specific interaction motifs to promote cancer progression.

## Materials and methods

### Protein purification and truncations

ETS proteins were purified as previously described [33]. All ETS genes were expressed from pET28a (EMD Biosciences, Darmstadt, Germany) with N‐terminal 6xHIS in *Escherichia coli* BL21 pRIL and purified using Ni‐NTA agarose resin (QIAGEN, Hilden, Germany). Protein concentrations were determined by electrophoresis on a 10% SDS/PAGE gel stained with Brilliant Blue R‐250 (Fisher, 6104‐59‐2) and compared to known BSA (Fisher, BP1605‐100, Hampton, NH, USA) standards. ETS proteins ERF and ETV3L did not express to sufficient levels to purify. Thus, only 26/28 ETS proteins were tested in these studies. Truncations of proteins were made *via* PCR amplification of the desired regions that were cloned into pET28a. These constructs were sequence‐verified, expressed in *Escherichia coli* BL21 pRIL, and purified as mentioned above.

p53 proteins (both wild‐type and mutants) were purified as in [34]. Briefly, the pET28a‐His‐p53 (and mutants) were transformed into *E. coli* Rosetta and selected for kanamycin resistance (50 μg·mL^−1^) on LB plates. Colonies were picked and grown overnight in 50 mL of LB with kanamycin in a shaker at 37 °C. The next day, 500 mL of LB with kanamycin was inoculated with the overnight culture until the OD_600_ = 0.6. The culture was transferred to a shaker at 25 °C, and 1 mm IPTG was added for 4 h. Thereafter, the bacteria were pelleted by centrifugation and stored in a −80 °C freezer. The pellet was thawed and resuspended in 50 mm NaH_2_PO_4_, 250 mm KCl, 0.05% IGEPAL, 1 mm dithiothreitol (DTT), and 10 mm imidazole, pH 8.0. The pellet was sonicated, and then debris was pelleted. The sample was loaded onto a Ni NTA column. The column was washed with lysis buffer in the absence of IGEPAL and the addition of 1 m KCL, then washed with 10 column volumes of lysis buffer without IGEPAL. p53 was eluted in PBS with 500 mm Imidazole, dialyzed into PBS pH 7.2 with 25% glycerol. His‐tag was removed by incubation with thrombin‐agarose, and any uncleaved protein was removed by exposure to Ni NTA. Protein concentration was determined and run on an SDS/PAGE gel and stained with Coomassie brilliant blue to confirm the size. Protein was aliquoted and stored at −80 °C. DNA binding of this batch of purified p53 was confirmed [35].

### Affinity pulldown assays with purified proteins

**Fig. 1:**
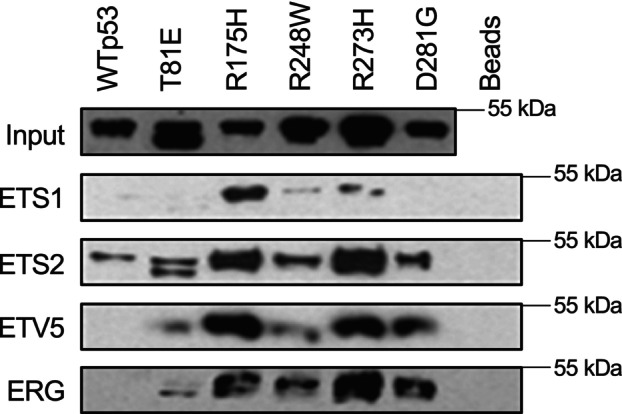
ETS proteins interact with mutant p53. Purified wild‐type p53 (WTp53), a phosphomimic mutant of p53 (T81E), or patient‐derived mutants of p53 (R175H, R248W, R273H, D281G) proteins were incubated with different purified ETS proteins (ETS1, ETS2, ETV5, and ERG) covalently fused to agarose beads. Following a wash, p53 was detected by immunoblot. Beads alone indicate ETS protein fused beads lacking p53.

**Fig. 2:**
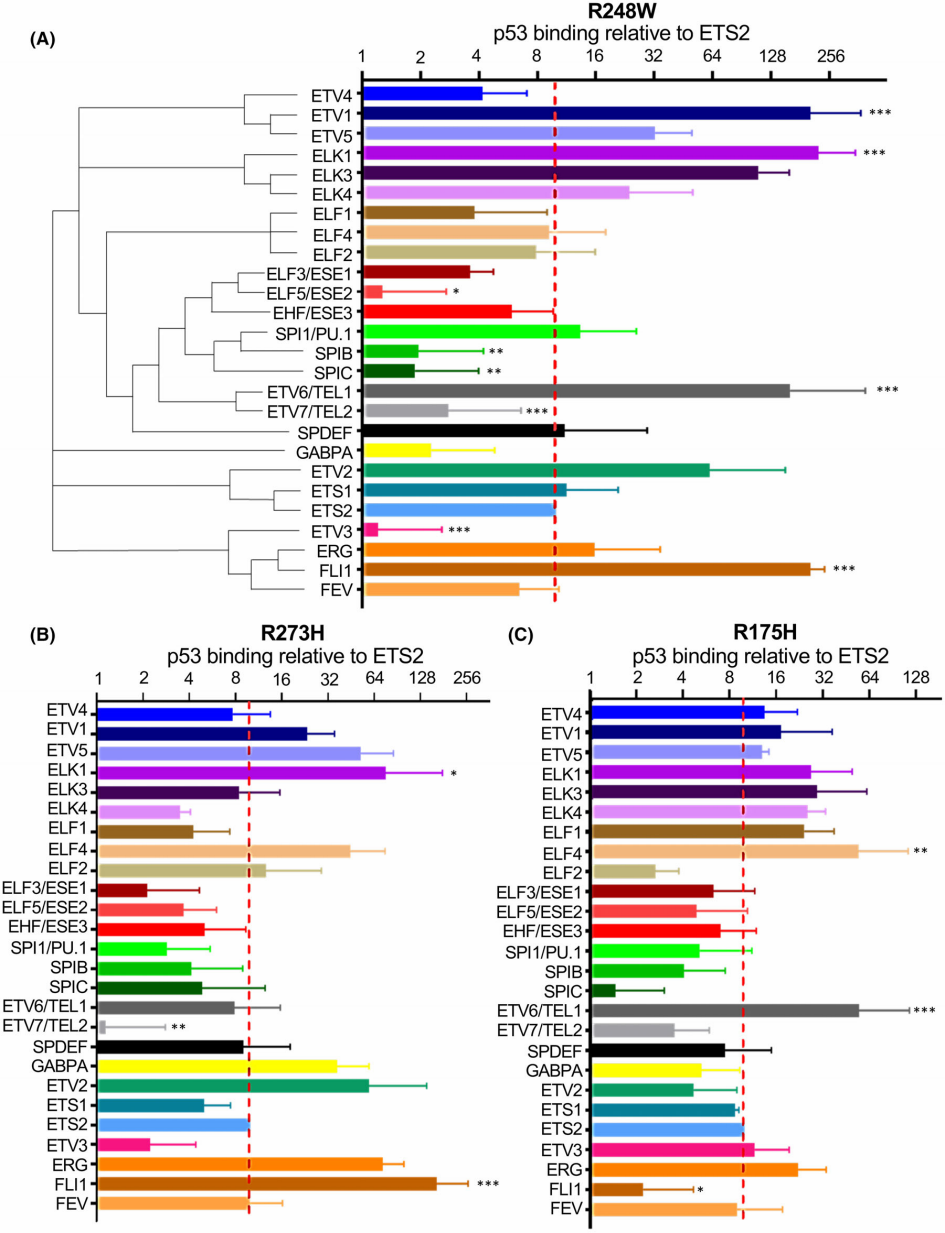
Numerous ETS family members interact with p53 mutants. The ETS family members, oriented both spatially and color‐wise by subfamily, and the amount of p53 binding relative to ETS2 for (A) R248W p53, (B) R273H p53, and (C) R175H p53. Each His‐ETS protein was bound to cobalt His‐isolation beads. Purified p53 that was retained on beads was measured by immunoblot and normalized to amount of the ETS bound and to the signal from ETS2 on the same blot ×10. *N* = 3 or more. Mean and SEM shown. ETS‐mutant p53 interaction ratios were compared to ETS2 interaction ratios by one‐way ANOVA. *P = 0.05, **P = 0.01, ***P ≤ 0.001.

**Fig. 3:**
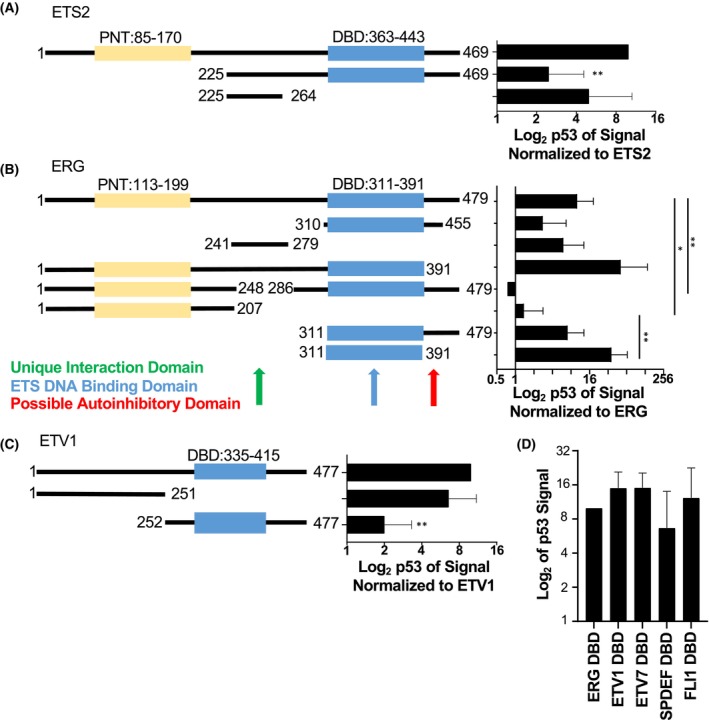
Strong interactors have two domains of interaction. (A) R248W p53 binding interaction with full‐length ETS2, a C‐terminal truncation of ETS2, or the 40‐amino acid region of interaction identified by Do et al. measured as in Fig. 2. Truncations were compared to full‐length ETS2 (×10). *P*‐value by ANOVA, **P ≤ 0.01, mean and SEM, *n* = 3. (B) R248W p53 binding interaction with full‐length ERG or various truncations of ERG measured as in Fig. 2, except normalized to full‐length ERG (×10). Indicated comparisons by ANOVA, *P ≤ 0.05, **P ≤ 0.01, mean and SEM, *n* = 3. (C) R248W p53 binding interaction with full‐length ETV1 or a N‐terminal truncation or a C‐terminal truncation measured as in Fig. 2 except normalized to full‐length ETV1 (×10) *via* ANOVA, **P ≤ 0.01, mean and SEM, *n* = 3. (D) R248W p53 binding interaction with the DNA binding domains (DBD) of ETS that displayed strong (ETV1 and FLI1), moderate (SPDEF and ERG), and weak (ETV7) interaction in Fig. 2. These determinations were based on statistical significance when compared to the ETS2 interaction; strong was significantly higher, weak was significantly lower, and moderate was not statistically different. Amino acids included in the DBD were ERG 311–391, ETV1 335–415, ETV7 224–305, SPDEF 249–332, and FLI1 281–361. Signal is normalized to the interaction with the ERG DNA binding domain (×10). Comparisons were made to the ERG DNA binding domain by ANOVA with no significant difference, mean and SEM, *n* = 3.

Affinity pulldown assays were performed with purified ETS proteins and purified p53 constructs. In Fig. 1 equal amount of either wild‐type p53 (WTp53), a phosphomimic mutant of p53 (T81E), or patient‐derived mutants of p53 (R175H, R248W, R273H, D281G) were incubated with purified ETS proteins (ETS1, ETS2, ETV5, and ERG) covalently fused to NHS‐activated agarose beads (ThermoFisher, Waltham, MA, USA). Following a wash with NP‐40 lysis buffer (50 mm Tris/HCl pH 7.4, 250 mm NaCl, 5 mm EDTA, 10 mm NaF, and 1% Nonidet P‐40), samples were boiled in SDS‐loading buffer, run on SDS/PAGE gels, and p53 was detected by immunoblot. In Figs 2 and 3, 5 μg of His‐tagged ETS protein was incubated with 300 μL of Binding Buffer (100 mm sodium phosphate pH 8.0, 600 mm NaCl and 0.02% Tween) and 50 μL of His‐tagged isolation and pulldown beads (ThermoFisher, 10104D) at 4 °C for 10 min. ETS‐conjugated beads were washed with 700 μL of Binding Buffer for two periods of 5 min to remove unbound protein. Bead bound proteins were blocked in 500 μL of NP‐40 lysis buffer and 10 μL of 10 mg·mL^−1^ BSA solution for 30 min. 30 ng of p53 (R248W, R175H, or R273H) was added to blocked ETS‐Bead conjugates and incubated at 4 °C for 30 min. Following incubation, samples were washed four times with 1 mL of NP‐40 lysis buffer. After washing, the proteins were eluted with SDS‐loading dye and electrophoresed on an SDS/PAGE gel and transferred to a nitrocellulose membrane. The membrane was stained with Ponceau (Fisher, BP103‐10) prior to immunoblotting for p53. Interaction strength was measured by a ratio of the ETS protein present determined by ponceau and the amount of bound p53 determined by immunoblot and comparing it to the ETS2‐mutant p53 interaction in the same blot. All ratios were plotted with the ETS2 interaction being set to 10.

### Cell lines, production of virus, viral transduction, and cell selection

VCaP (RRID:CVCL_2235) cells were obtained from ATCC and maintained according to manufacturer protocols. Briefly, cells were grown in Dulbecco's Modified Eagle Medium (DMEM) (Corning, Corning, NY, USA) supplemented with 10% Fetal Bovine Serum (Sigma, St. Louis, MO, USA) and 1% Penicillin–Streptomycin (ThermoFisher). They were maintained at 37 °C with 5% CO_2_. HEK293T (RRID:CVCL_0063) cells that were used to produce lentivirus were also grown in the same conditions as the VCaP cells according to the manufacturer protocols. Lentivirus shRNA for control and ERG knockdown were produced as described previously [36]. Briefly, HEK293T cells were transfected with the packaging plasmids pMDLg/pRRE (Addgene, 12253, Watertown, MA, USA), pRSV‐Rev (Addgene, 12253), and pMD2.G (Addgene, 12259) and the pLKO.1 cloning vector (Addgene, 8453) that had been cloned to include the specific shRNA sequences (Table S1). Cells were transduced with the appropriate virus or mock virus and selected with puromycin after 24 h at a cell line‐dependent concentration. Media and puromycin were replaced every 2–3 days until all the cells in the mock plate were dead and those transduced with virus were surviving with selection. At that time, cells were maintained as stated above with the addition of puromycin to keep selection pressure.

All cell lines were authenticated using PowerPlex 16HS Assay with > 80% match to eight core STR loci within 3 years. Cell lines in use are tested for mycoplasma every 4 months using a PCR mycoplasma detection kit (AbCam, Cambridge, UK).

### Immunoblotting

Whole cell extracts were generated from confluent plates of equivalent cell number. Lysates were then subjected to electrophoresis on a 10% SDS/PAGE gel, transferred to nitrocellulose membrane (Bio‐Rad, 1620115, Hercules, CA, USA), and blocked in 5% non‐fat milk in TBST. Primary antibodies were diluted at 1 : 1000 in blocking buffer and allowed to incubate overnight. Antibodies were ERG (BioCare Medical, CM421C, Pacheco, CA, USA), H3 (Cell Signaling, C36B11, Danvers, MA, USA), ETV1 (Gift from Shyh‐Han Tan [37]), FLAG (F1804, Sigma), p53 (DO‐1) (sc‐126, Santa Cruz Biotechnology, Dallas, TX, USA), GAPDH (47724, Santa Cruz Biotechnology), and MDM2 (2A10, Sigma). Secondary antibodies (anti‐Rabbit or anti‐Mouse, Cytiva, Buckinghamshire, UK, 45‐000‐682 or 45‐000‐692 respectively) were diluted in blocking buffer at a concentration of 1 : 12 500 and incubated for 1 h. Following washing with TBST, membranes were developed with ECL (Pierce, PI34095).

### Chromatin immunoprecipitation sequencing

ChIP was previously described [38]. Briefly, all cells were crosslinked *via* 1% v/v formaldehyde (Fisher Scientific, BP531‐25) for 15 min followed by a 5‐min 2 m Glycine quench. Cells were then lysed and sonicated for 3 min (30 s On/Off) using a Diagenode, Biorupter Pico. The nuclear lysates were rotated with the specific antibody for 4 h at 4 °C and washed prior to crosslink reversal. The DNA was isolated *via* phenol/chloroform extraction and purified for sequencing. The antibodies used were p53 (DO‐1) (sc‐126, Santa Cruz Biotechnology), and they were conjugated to sheep anti‐mouse Dynabeads (11202D, Invitrogen).

Library preparation was previously described [39]. Briefly, at least three ChIPs were pooled together for each antibody prior to library preparation and samples sonicated using a Diagenode Biorupter to ~ 150 bps. Fragment end repair was accomplished using Klenow DNA polymerase, T4 DNA polymerase, and T4 DNA ligase (New England BioLabs, Ipswich, MA, USA) and QIAQuick PCR Purification (Qiagen). TruSeq adaptors (Illumina, San Diego, CA, USA) were ligated *via* T4 DNA Ligase (New England BioLabs). Products were size selected on a 2% Agarose gel and bead cleanup was performed with AMPure XP beads (Beckman Coulter, Brea, CA, USA). Purified libraries were multiplexed and sequenced *via* single reads using a NextSeq75 flow cell on an Illumina NextSeq500.

Reads were aligned, and peaks were called as previously described [40]. Briefly, alignment of reads was performed using bowtie2 (hg19) with the Blacklist region removed *via* bedtools. Duplicates were also removed *via* Picard‐2.14.0. MACS2 was used to call peaks with a *P*‐value of < 0.001. Ad hoc analysis was done to only utilize peaks with a *q*‐value of < 0.001. Nearest genes were determined by nearest transcription start site and were called *via* HOMER after liftover to hg38 on the ucsc website. Bedtools intersect command was used to determine overlapping peaks. Peaks were visualized *via* the interactive genome viewer (IGV). The ERG ChIP‐Seq and input raw files (GSE28950) were downloaded from the NCBI Geo Database and the reads aligned and peaks were called using our established pipeline described above.

## Results

### Mutant p53 binds four ETS family members better than wt p53

ETS family members ETS1, ETS2, ETV5, and ERG were purified, covalently bound to agarose beads, and exposed to purified wt p53, a phosphomimetic p53 mutant (T81E), and p53 mutations commonly found in cancer (R175H, R248W, R273H, and D281G) (Fig. 1). Consistent with a previous report [19], ETS2 could bind wt p53, yet binding of ETS2 was more evident for p53 with gain‐of‐function mutations found in tumors. ETS1, ETV5, and ERG‐bound patient‐identified p53 mutations, but did not bind wt p53. The T81 phosphomimetic p53 mutation showed stronger binding to ETV5 and ERG compared to wt p53. A previous study indicates that phosphorylation at this site can mimic conformational changes caused by patient mutations [34].

### Mutant p53 binding is widespread in the ETS family, but interaction strength is diverse

To compare binding of individual mutant p53 proteins across the ETS family, all 28 human ETS genes were cloned with N‐terminal 6xHis tags and expressed in *E. coli*. 26 of the 28 ETS proteins successfully expressed and were purified. Each of these were then bound to magnetic cobalt beads and exposed to a purified mutant p53. Gain‐of‐function R248W, R273H, and R175H p53 were tested because these are three of the most common mutations and comprise both DNA binding (R248 and R273) and conformational (R175) mutants [41]. Binding to each ETS was determined by immunoblotting, normalized to the amount of ETS protein on the membrane, then normalized to the ETS2 binding ratio from the same gel (Fig. 2). Representative images of individual experiments and purified proteins are in Fig. S1. All ETS proteins showed some degree of binding to each mutant p53 protein compared to bead alone controls (Fig. 2). 12 ETS proteins bound R248W p53 more strongly than ETS2. Similar patterns were observed for R273H and R175H, with seven and eight of the same ETS proteins binding better than ETS2, respectively (Fig. 2 and Table 1). Four ETS proteins, ETV1, ETV5, ELK1, and ERG, bound all three p53 mutants better than ETS2. There were several notable differences in interaction between p53 mutants. R175H binding was more consistent across the ETS family than R248W or R273H, but R175H displayed a decreased interaction with FLI1 and ELF2. GABPA bound strongly to R273H, weakly to R248W, and moderately to R175H. ETS proteins can be sorted into subfamilies of up to three members based on homology in the DNA binding domain (Fig. 2A). Mutant p53 binding strength did not sort by subfamily. For example, ETV6 displayed moderate to strong binding to all three p53 mutants, but close homolog ETV7 bound weakly to all three. Similarly, R248W p53 strongly bound SPI1, but SPIB and SPIC binding was weak

**Table 1:** Classification of ETS proteins by interaction with mutant p53 compared to ETS2. ETS that bind indicated p53 mutant with stronger (+), weaker (−), or the same (=) signal as ETS2. Significance (*) and classified as a strong interactor in Fig. 6 (^a^) are indicated.

ETS protein	R248W	R273H	R175H
ETV1^a^	+*	+	+
ETV5^a^	+	+	+
ELK1^a^	+*	+*	+
ERG^a^	+	+	+
ETS2^a^	=	=	=
ETV2^a^	+	+	−
FLI1^a^	+*	+*	−*
ELK3^a^	+	−	+
ELK4^a^	+	−	+
ETV6^a^	+*	−	+*
ELF4	−	+	+*
SPI1^a^	+	−	−
SPDEF^a^	+	−	−
ETS1^a^	+	−	−
ELF2	−	+	−
GABPA	−	+	−
ETV4	−	−	+
ELF1	−	−	+
ETV3	−*	−	+
ELF3	−	−	−
ELF5	−*	−	−
EHF	−	−	−
SPIB	−*	−	−
SPIC	−*	−	−
ETV7	−*	−*	−
FEV	−	−	−

### ETS family members that exhibit strong interactions with mutant p53 have two sites of interaction

Do et al. [19] mapped a 40 amino acid region of ETS2 as necessary for the interaction with R175H p53 but did not test if this region was sufficient. We purified this 40 amino acid region of ETS2 and found it was sufficient to bind R248W p53 (Fig. 3A, Fig. S2A,B). There was no apparent homology between this 40 amino acid sequence and any region of any other ETS protein. In fact, this region does not have similarity even with the closest ETS2 homolog, ETS1, which interacts similarly with mutant p53 (Fig. 2). To map an interaction domain on a second ETS protein, several truncations and deletions of ERG were purified and tested for binding to R248W p53 (Fig. 3B, Fig. S2C). Deletion of the region between 248 and 286 resulted in loss of mutant p53 interaction. A similar 39 amino acid region of ERG (241–279) was sufficient for interaction with R248W p53 (Fig. 3B). Despite the similarity in the size and relative location of the 241–279 region of ERG to the mutant p53 interacting region of ETS2, there was no obvious sequence similarity. Surprisingly, two fragments containing the ETS DNA binding domain but lacking the region from 248 to 286 also bound R248W p53 (310–455 and 311–479). Therefore, the isolated ETS DNA binding domain (311–391) was tested and found to be sufficient for binding R248W p53 (Fig. 3B). These results indicate that ERG has two distinct mutant p53 interaction domains, one between the ETS DNA binding domain and the PNT domain, and the DNA binding domain alone.

It is unclear why the ERG construct lacking the region between residues 248 and 286 showed no mutant p53 binding despite including the ETS DNA binding domain, which can interact with mutant p53 (Fig. 3B). We hypothesize that this is due to autoinhibition of the ETS DNA binding domain interaction with mutant p53 by a region of ERG from 391 to 455. Fragments that include the DNA binding domain and 391–455 (310–455 and 311–479) displayed less binding to mutant p53 than the isolated DNA binding domain (311–391). Further, full‐length ERG had less binding than an N‐terminal truncation (1–391) (Fig. 3B). Together these trends suggest that the ERG DNA binding domain association with mutant p53 is inhibited by amino acids 391–455. Domains that autoinhibit DNA binding are known to flank the ERG DNA binding domain [42]. Similar autoinhibition of mutant p53 binding indicates there could be a mechanism whereby these regions structurally block both DNA and mutant p53 binding.

A common interaction with the conserved ETS DNA binding domain would explain the ability of mutant p53 to bind the entirety of the ETS family. However, strong mutant p53 interactors ETS2 and ERG have additional interaction domains outside of the ETS DNA binding domain (Fig. 3A,B). Therefore, we hypothesized that all isolated ETS DNA binding domains bind mutant p53 equally, but that full‐length ETS proteins that strongly bind mutant p53 have a second interaction domain. To test this, N‐terminal and C‐terminal halves of the strong p53 interactor ETV1 were purified, where the ETS DNA binding domain is in the C‐terminal half. N‐terminal ETV1 bound R248W p53 more strongly than C‐terminal ETV1 (Fig. 3C), indicating the presence of an interaction domain in the ETV1 N terminus. The significantly weaker interaction with the C‐terminal half of ETV1 also suggests autoinhibition of the DNA binding domain interaction, similar to ERG. To test the hypothesis that differences in binding strength do not arise from the DNA binding domain interaction, isolated ETS DNA binding domains of strong p53 R248W interactors (ETV1 and FLI1), moderate interactors (ERG and SPDEF), and a weak interactor (ETV7) were purified and compared for binding to R248W p53 (Fig. 3D, Fig. S2D). Despite significant differences in interactions between the full‐length proteins (Fig. 2), the isolated DNA binding domains of these ETS proteins showed similar binding. Together these findings are consistent with a model in which the presence or absence of a second interaction region outside of the ETS DNA binding domain dictates whether an ETS protein will strongly bind mutant p53.

### Identification of an amino acid motif that promotes interaction with mutant p53

**Fig. 4:**
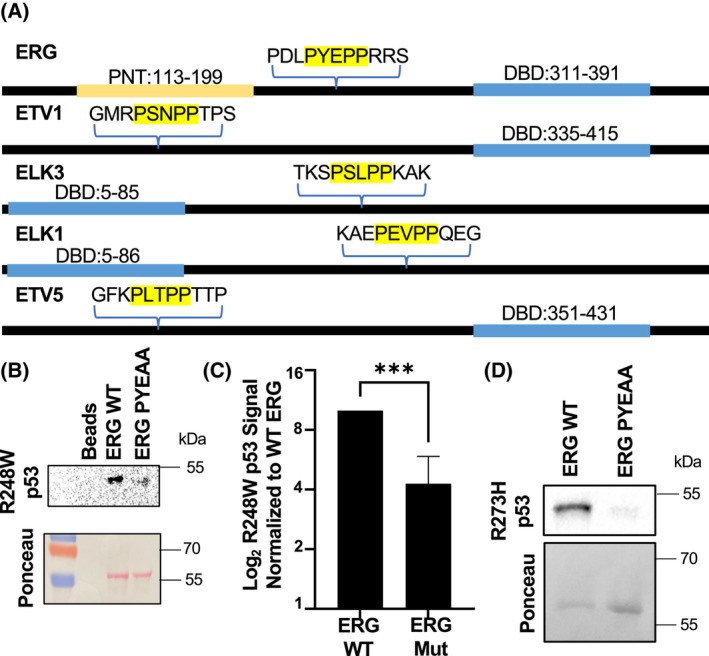
Alteration of the unique ERG mutant p53 interaction site reduces interaction strength. (A) Schematic of all tested ETS proteins with a PXXPP motif with at least one polar residue. (B) Representative image of the R248W p53 binding interaction with wt (PYEPP) ERG or mutant (PYEAA) ERG. (C) Quantification of the p53 signal normalized to wt ERG (×10). Comparison by *t*‐test, ***P = 0.0001, mean and SEM, *n* = 3. (D) Similar to (B), ERG or PYEAA ERG were immobilized on beads and equal quantities of purified R273H p53 added and washed. Ponceau indicates amount of purified ERG and immunoblot shows p53 binding.

The regions of ERG and ETV1 outside of the DNA binding domains that interacted with mutant p53 are disordered regions with many proline residues. Examination of these regions identified a PXXPP motif where at least one of the amino acids between the prolines is polar. Only five of the 26 tested ETS proteins have this motif: ERG, ELK1, ELK3, ETV1, and ETV5. Strikingly, all four of the ETS proteins that were strong interactors (better than ETS2) across all p53 mutants (ERG, ELK1, ETV1, and ETV5; Table 1) harbor this motif (Fig. 4A). The only other tested ETS protein with this motif, ELK3, strongly interacted with two of the three p53 mutants. To test the importance of PXXPP, we mutated the motif in ERG from PYEPP to PYEAA, purified the mutant protein, and tested mutant p53 binding. The mutation of PP to AA resulted in reduced interaction of ERG with both R248W p53 (Fig. 4B,C) and R273H p53 (Fig. 4D). This suggests that the PXXPP motif is important for the interaction with mutant p53 in these five ETS proteins.

### Loss of ERG alters mutant p53 association with the genome

**Fig. 5:**
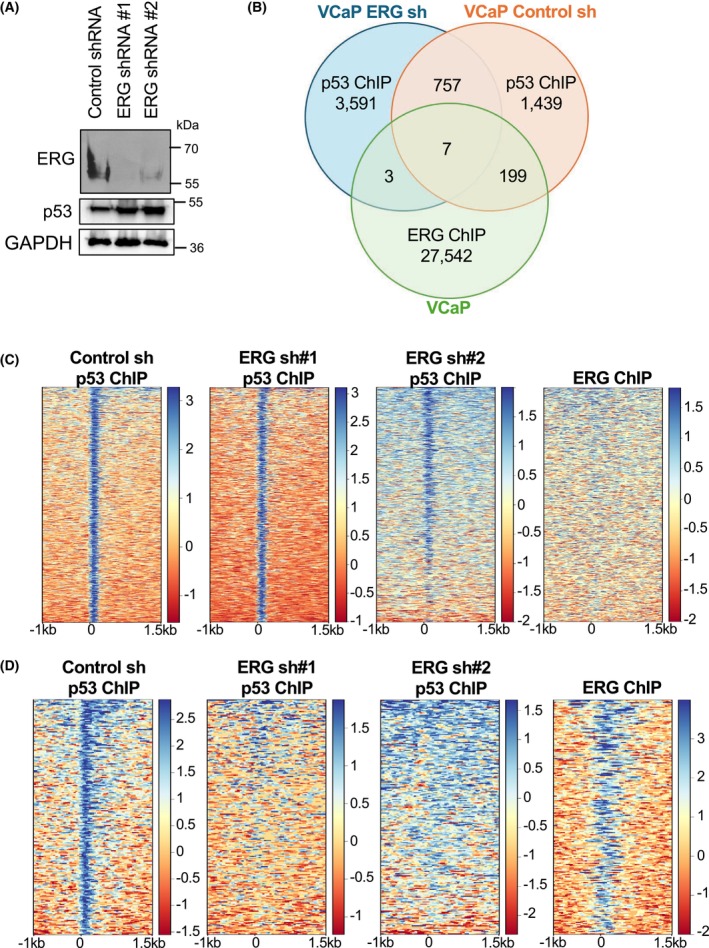
Identification of ERG‐dependent mutant p53 bound regions. (A) Immunoblots of ERG and p53 in the VCaP cell line with control shRNA targeting luciferase, or one of two independent shRNAs targeting ERG. GAPDH is a loading control. (B) Venn diagram showing the overlapping bound regions for the p53 ChIP‐Seq in VCaP cells with luciferase (control) shRNA (sh) or ERG shRNA in comparison to a previously published ERG ChIP‐Seq in VCaP cells. 199 overlaps of ERG with p53 specific to control knockdown compared to 3 overlaps with p53 specific to ERG knockdown is significant, *P* < 0.0001 by Fisher's exact test. (C) Heatmaps centered on the overlapping mutant p53‐bound regions between control and ERG knockdown. (D) Heatmaps centered on the overlapping regions bound by both mutant p53 and ERG in control cells.

To test if an ETS transcription factor that strongly interacts with R248W p53 *in vitro* can recruit mutant p53 in cells, p53 genomic occupancy was measured in the VCaP prostate cancer cell line. VCaP cells have a *TMPRSS2/ERG* gene rearrangement resulting in overexpression of ERG, harbor R248W mutant p53, and have a deletion in the other allele encoding p53 [43, 44]. The *TMPRSS2/ERG* gene rearrangement is the most common mutation in prostate cancer, found in about 50% of patient cases [45]. Two independent shRNAs reduced ERG expression compared to a control shRNA targeting luciferase (Fig. 5A). ChIP‐seq mapped p53 bound regions in the presence and absence of ERG knockdown. There were 2402 called p53 peaks in control shRNA cells, 4358 called p53 peaks in ERG shRNA cells, and 764 of these p53 bound regions overlapped (Fig. 5B). p53‐bound regions were compared with ERG‐bound regions from previously published ERG ChIP‐Seq in VCaP cells (GSE28950 [46]). ERG‐bound regions had very little overlap with p53‐bound regions in ERG knockdown cells (3, 0.07%), or with p53‐bound regions found in both control and knockdown cells (7, 0.16%). The majority of ERG/p53 overlap was observed in regions bound by p53 in control cells, but not in ERG knockdown cells (199, 8%) (Fig. 5B). These co‐occupied regions tended to be in promoters or gene bodies, regions rich in transcriptional control elements (Fig. S3A). Co‐occupied regions were also enriched for an ETS binding motif (Fig. S3B). To validate these findings, ChIP‐seq of p53 was performed in VCaP cells with ERG knocked down with a second independent shRNA. In this replicate, p53 occupancy was confirmed in ERG‐independent regions (Fig. 5C), and p53 was again lost from ERG co‐bound regions (Fig. 5D). This suggests that there is a subset of mutant p53 genomic occupancy dependent on ERG. Genes near the 199 ERG/p53 co‐occupied regions were compared to published gene expression changes when ERG is knocked down in VCaP cells [47], or a comparison of ERG‐positive p53‐null murine prostate tumors to ERG‐positive p53‐mutant tumors [25]. *CRISP3* and *TYMS* displayed ERG‐dependent p53 binding (Fig. S3C), were significantly downregulated upon ERG knockdown [47], and significantly upregulated in ERG/p53‐mutant tumors compared to ERG/p53‐null tumors [25]. Both *CRISP3* and *TYMS* are associated with prostate cancer progression [48, 49] suggesting that ERG/p53 regulation could promote oncogenic phenotypes through these target genes.

### *TP53* mutation correlates with upregulation of ETS in ovarian cancer

**Fig. 6:**
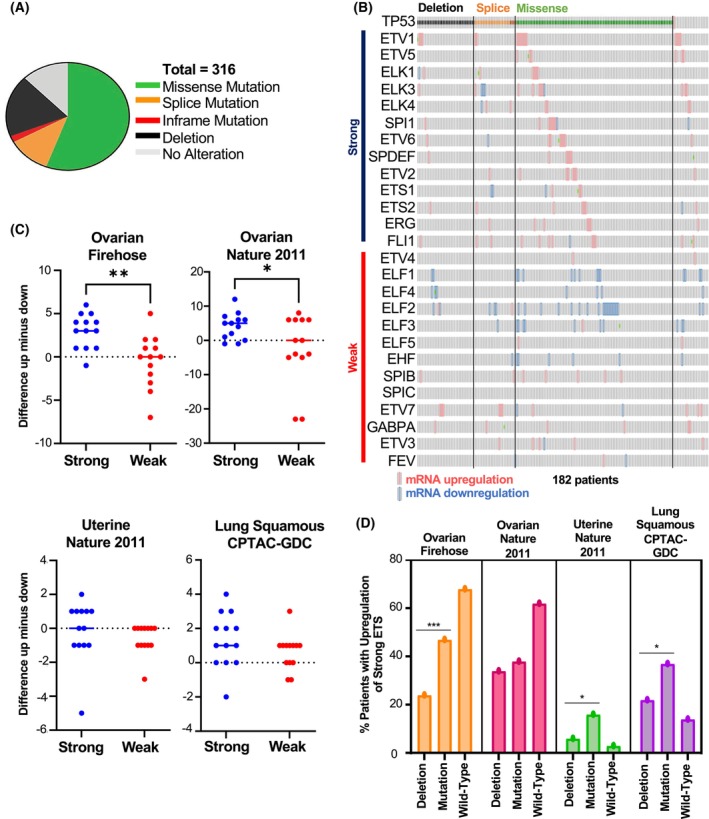
Upregulation of strong ETS among patients with p53 mutation. (A) p53 mutations in the TCGA Firehose Legacy Ovarian Serous Cystadenocarcinoma dataset. (B) The TCGA Firehose Legacy Ovarian Serous Cystadenocarcinoma dataset stratified by p53 status. Strong interacting ETS (ETS2 and higher interaction with R248W) and weak interacting ETS relative mRNA level and TP53 mutation status indicated. Mutations in p53 color coded as in (A). For ETS mRNA: Red, upregulated; Blue, downregulated; Green, mutation. (C) Quantification of data in (B) and three additional datasets as indicated. Each dot is an ETS gene and difference was calculated by number of tumor samples with upregulation minus number with downregulation only within tumors with missense p53 mutation. Comparisons by *t*‐test, *P = 0.01 and **P = 0.001. (D) Summary data for the percentage of patients with upregulation of strong ETS co‐occurring with either mutant p53, deleted p53, or wild‐type p53 (WT) across indicated datasets. Comparison by Fisher's exact test, ***P = 0.001, *P = 0.05.

If recruitment of mutant p53 to the genome by an ETS protein provides an advantage to a cancer cell, higher expression of strong mutant p53 interactors and lower expression of weak mutant p53 interactors might be selected for during cancer evolution. To test if ETS proteins that strongly interact with mutant p53 are upregulated in human cancer, publicly available data were compared for several cancer types. The TCGA Firehouse Legacy Ovarian Serous Cystadenocarcinoma dataset (GDAC Firehose, Broad Institute, UID: 10485) was tested because of high levels of p53 mutations (Fig. 6A). The mutants tested in Fig. 2 (R175H, R248W, and R273H) were among the most common sites of mutation and account for 21% of total p53 mutations (Fig. S4). ETS proteins were classified as strong mutant p53 binders if they bound R248W p53 equal to or better than ETS2 and as weak if the binding was less than ETS2 (Fig. 2A). A pattern emerged of upregulation of strong ETS and downregulation or no change in weak ETS (Fig. 6B). Upregulation showed some level of mutual exclusivity, but downregulation did not. Changing the order by plotting weak binders first did not alter this pattern. Plotting the difference in the number of tumors with upregulation versus downregulation indicated a significant difference between expression of strong and weak mutant p53 binders (Fig. 6C). This same pattern was not observed in multiple other tumor types, for example, lung adenocarcinoma (Fig. S5). However, the pattern was observed in a second ovarian serous cystadenocarcinoma dataset [50] and a similar, but weaker, trend occurred in uterine carcinosarcoma and lung squamous carcinoma datasets (Fig. 6C). When the ovarian cancer, uterine cancer, and lung squamous datasets were further stratified for the upregulation of strong ETS according to p53 status, there was an increased fraction of tumors with upregulation in the patients with mutant p53 compared to deletion of p53 (Fig. 6C), suggesting that this trend is selected in tumors with p53 mutation specifically. Unexpectedly, there is also a high percentage of patients with upregulation of strong ETS in the small subset of patients with wt p53 in the two ovarian cancer datasets (Fig. 6C), but not in the uterine or lung squamous cell carcinoma dataset. This could be due to error in the p53 status due to heterogeneity. However, it could also suggest a gain of function role for wt p53 due to phosphorylation, as Fig. 1 indicates increased ETS protein interaction with phosphomimetic T81E p53. Overall, these results suggest that there is a correlation between ETS that were identified as strong interactors and p53 mutation status in cancer patients, but only in certain cancer types.

## Discussion

Our findings indicate that mutant p53 has a biochemical gain‐of‐function in the ability to bind to multiple ETS family transcription factors. Analysis of patient data indicates that these same ETS that bind mutant p53 are upregulated in ovarian cancer with mutant p53 compared to tumors with *TP53* deletion. In ovarian cancer, p53 mutation is implicated in particularly poor prognosis [11]. In prostate cancer, p53 mutation is generally a late event that occurs in response to antiandrogen treatment and promotes castration resistance [15, 51, 52]. *CRISP3* and *TYMS* are interesting potential target genes where mutant p53 binding is dependent on ERG in prostate cancer. *CRISP3* was previously identified as a gene overexpressed specifically in ERG‐positive prostate cancer and correlated with high Gleason score [53]. Further, CRISP3 can promote prostate cancer progression in a mouse model [48]. TYMS encodes thymidylate synthase, which plays a key role in pyrimidine metabolism. A recent study found that increased pyrimidine metabolism correlates with p53 mutation in prostate cancer [54].

These findings suggest that multiple different ETS factors could recruit mutant p53 to the genome, allowing mutant p53 to activate target genes to promote oncogenic phenotypes. All ETS interacted with mutant p53 to some extent, but there was variability across p53 mutations and across ETS proteins. A common mutant p53 interaction interface was the ETS DNA binding domain. However, ETS that strongly bound mutant p53 had a second interaction site outside of the DNA binding domain that contained a PXXPP motif. Ovarian cancer patient data demonstrated that there is upregulation of ETS that strongly bound mutant p53 in patients that also harbor a mutation in p53. This suggests that changes in the relative ratio of ETS proteins is selected for in tumors with mutant p53. It is possible that reduced expression of ETS proteins that bind mutant p53 weakly could allow more genomic binding of ETS proteins that bind mutant p53 strongly, providing the same oncogenic advantage as increasing expression of strong binders. Our genomic data find that genomic regions where mutant p53 binding overlaps with ERG are preferentially lost when ERG is knocked down (Fig. 5B). However, the majority of ERG‐bound regions did not also bind p53. It is unclear what might be different about a region where p53 binding is ERG‐dependent, and regions where ERG does not recruit p53. Wild‐type transcriptional co‐activators such as p300 require interaction with multiple transcription factors bound across a regulatory sequence [55]. It is possible that mutant p53 recruitment is similar.

In the p53 field, it is frequently debated whether the phenotypes associated with mutation are due to the loss of its wild‐type tumor suppressor function, dominant‐negative effects, or a gain‐of‐function associated with the mutation [17]. All could be true. Our data focus on the ETS family and aligns with the gain‐of‐function activity for mutations of p53 as we have shown a biochemical gain‐of‐function, with mutant p53 binding ETS proteins better than wild‐type p53 (Fig. 1). A recent study showing that the effects of mutant p53 are due to loss of function utilized a variety of cell lines to demonstrate that the deletion of p53 had no effect on proliferation or response to chemotherapeutics [10]. However, the cancer model systems used were ones where we did not identify any correlation between strong ETS and mutant p53. The correlation of strong ETS and mutant p53 specifically occurred in the related diseases of serous ovarian cancer and uterine cancer. We did not test correlation in prostate cancer, as these results would be dominated by ERG and ETV1 gene rearrangements. Both ERG and ETV1 were among the strongest ETS interactors with mutant p53, suggesting a role in this disease as well. Our findings suggest a cancer‐type‐dependent role for gain‐of‐function p53.

Upregulation of ETS proteins with strong mutant p53 interactions was observed more often in cancers with p53 mutations than in cancers with p53 deletion in three of the four datasets analyzed in Fig. 6D. In both of the ovarian cancer datasets, upregulation of strong interacting ETS was even higher in cancers with wild‐type p53. However, this correlation was not significant due to the very small number of cancers with wild‐type p53, and this was not observed in the uterine or lung squamous cell datasets. Despite this, these data suggest that there could be some interesting correlation between wild‐type p53 and ETS factors specifically in ovarian cancer.

These studies suggest a critical role for ETS transcription factors in gain‐of‐function mutant p53. Thus, there is the potential for small molecule inhibitors to prevent this protein–protein interaction. Mutant p53 has been identified as an important driver of cancer, but to date there have been no successful small molecules that target mutant p53 specifically [41]. This could be attributed to the crucial nature of p53 to normal cellular function [17]. Current targeting methods include trying to restore wild‐type p53 function, targeting mutant p53 for degradation, and utilizing immunotherapies, although some of these need more study or are poorly understood [41]. Targeting interacting partners of mutant p53 could provide specificity by not affecting wild‐type p53 function. The potential to target the interaction between ETS and mutant p53 could result in additional powerful tools for clinicians to treat patients with overexpression of ETS and specific mutations in p53.

## Author contributions

SAM and PCH conceived the study. PCH supervised the study. SAM and PCH designed experiments. SAM, NFD, KMM, SCM, AEK, and LDM performed experiments. SAM and PCH wrote the manuscript. PCH and LDM edited the manuscript.

## Supporting information


**Fig. S1.** Representative images of purified proteins and binding assay.
**Fig. S2.** ETS truncations interaction with mutant p53.
**Fig. S3.** Analysis of p53 genomic binding.
**Fig. S4.** Patient mutation frequency for all p53 mutations that occur more than once across the Ovarian TCGA Firehose Legacy dataset.
**Fig. S5.** Lung adenocarcinoma dataset.
**Table S1.** shRNA sequences.

## Data Availability

The data underlying this article are available in NCBI Gene Expression Omnibus (GEO) at https://www.ncbi.nlm.nih.gov/geo/ and can be accessed with GSE270925.

## References

[1] Song H , Hollstein M and Xu Y (2007) p53 gain‐of‐function cancer mutants induce genetic instability by inactivating ATM. Nat Cell Biol 9, 573–580. 17417627 10.1038/ncb1571

[2] Zilfou JT and Lowe SW (2009) Tumor suppressive functions of p53. Cold Spring Harb Perspect Biol 1, a001883. 20066118 10.1101/cshperspect.a001883 PMC2773645

[3] Mantovani F , Collavin L and Del Sal G (2019) Mutant p53 as a guardian of the cancer cell. Cell Death Differ 26, 199–212. 30538286 10.1038/s41418-018-0246-9 PMC6329812

[4] Rucker FG , Schlenk RF , Bullinger L , Kayser S , Teleanu V , Kett H , Habdank M , Kugler CM , Holzmann K , Gaidzik VI *et al*. (2012) TP53 alterations in acute myeloid leukemia with complex karyotype correlate with specific copy number alterations, monosomal karyotype, and dismal outcome. Blood 119, 2114–2121. 22186996 10.1182/blood-2011-08-375758

[5] Xu Y (2008) Induction of genetic instability by gain‐of‐function p53 cancer mutants. Oncogene 27, 3501–3507. 18223686 10.1038/sj.onc.1211023

[6] Freed‐Pastor WA , Mizuno H , Zhao X , Langerod A , Moon SH , Rodriguez‐Barrueco R , Barsotti A , Chicas A , Li W , Polotskaia A *et al*. (2012) Mutant p53 disrupts mammary tissue architecture via the mevalonate pathway. Cell 148, 244–258. 22265415 10.1016/j.cell.2011.12.017 PMC3511889

[7] Schulz‐Heddergott R , Stark N , Edmunds SJ , Li J , Conradi LC , Bohnenberger H , Ceteci F , Greten FR , Dobbelstein M and Moll UM (2018) Therapeutic ablation of gain‐of‐function mutant p53 in colorectal cancer inhibits Stat3‐mediated tumor growth and invasion. Cancer Cell 34, 298–314.e297. 30107178 10.1016/j.ccell.2018.07.004 PMC6582949

[8] Alexandrova EM , Yallowitz AR , Li D , Xu S , Schulz R , Proia DA , Lozano G , Dobbelstein M and Moll UM (2015) Improving survival by exploiting tumour dependence on stabilized mutant p53 for treatment. Nature 523, 352–356. 26009011 10.1038/nature14430 PMC4506213

[9] Dibra D , Moyer SM , El‐Naggar AK , Qi Y , Su X and Lozano G (2023) Triple‐negative breast tumors are dependent on mutant p53 for growth and survival. Proc Natl Acad Sci U S A 120, e2308807120. 37579145 10.1073/pnas.2308807120 PMC10450424

[10] Wang Z , Burigotto M , Ghetti S , Vaillant F , Tan T , Capaldo BD , Palmieri M , Hirokawa Y , Tai L , Simpson DS *et al*. (2024) Loss‐of‐function but not gain‐of‐function properties of mutant TP53 are critical for the proliferation, survival, and metastasis of a broad range of cancer cells. Cancer Discov 14, 362–379. 37877779 10.1158/2159-8290.CD-23-0402 PMC10850947

[11] Cole AJ , Dwight T , Gill AJ , Dickson KA , Zhu Y , Clarkson A , Gard GB , Maidens J , Valmadre S , Clifton‐Bligh R *et al*. (2016) Assessing mutant p53 in primary high‐grade serous ovarian cancer using immunohistochemistry and massively parallel sequencing. Sci Rep 6, 26191. 27189670 10.1038/srep26191 PMC4870633

[12] Kang HJ , Chun SM , Kim KR , Sohn I and Sung CO (2013) Clinical relevance of gain‐of‐function mutations of p53 in high‐grade serous ovarian carcinoma. PLoS One 8, e72609. 23967324 10.1371/journal.pone.0072609 PMC3742716

[13] Tuna M , Ju Z , Yoshihara K , Amos CI , Tanyi JL and Mills GB (2020) Clinical relevance of TP53 hotspot mutations in high‐grade serous ovarian cancers. Br J Cancer 122, 405–412. 31780779 10.1038/s41416-019-0654-8 PMC7000721

[14] Nyquist MD , Corella A , Coleman I , De Sarkar N , Kaipainen A , Ha G , Gulati R , Ang L , Chatterjee P , Lucas J *et al*. (2020) Combined TP53 and RB1 loss promotes prostate cancer resistance to a Spectrum of therapeutics and confers vulnerability to replication stress. Cell Rep 31, 107669. 32460015 10.1016/j.celrep.2020.107669 PMC7453577

[15] Robinson D , Van Allen EM , Wu YM , Schultz N , Lonigro RJ , Mosquera JM , Montgomery B , Taplin ME , Pritchard CC , Attard G *et al*. (2015) Integrative clinical genomics of advanced prostate cancer. Cell 161, 1215–1228. 26000489 10.1016/j.cell.2015.05.001 PMC4484602

[16] Bieging KT , Mello SS and Attardi LD (2014) Unravelling mechanisms of p53‐mediated tumour suppression. Nat Rev Cancer 14, 359–370. 24739573 10.1038/nrc3711 PMC4049238

[17] Liebl MC and Hofmann TG (2021) The role of p53 signaling in colorectal cancer. Cancers (Basel) 13, 2125. 33924934 10.3390/cancers13092125 PMC8125348

[18] Moon SH , Huang CH , Houlihan SL , Regunath K , Freed‐Pastor WA , Morris JPt , Tschaharganeh DF , Kastenhuber ER , Barsotti AM , Culp‐Hill R *et al*. (2019) p53 represses the mevalonate pathway to mediate tumor suppression. Cell 176, 564–580.e519. 30580964 10.1016/j.cell.2018.11.011 PMC6483089

[19] Do PM , Varanasi L , Fan S , Li C , Kubacka I , Newman V , Chauhan K , Daniels SR , Boccetta M , Garrett MR *et al*. (2012) Mutant p53 cooperates with ETS2 to promote etoposide resistance. Genes Dev 26, 830–845. 22508727 10.1101/gad.181685.111 PMC3337457

[20] Zhu J , Sammons MA , Donahue G , Dou Z , Vedadi M , Getlik M , Barsyte‐Lovejoy D , Al‐awar R , Katona BW , Shilatifard A *et al*. (2015) Gain‐of‐function p53 mutants co‐opt chromatin pathways to drive cancer growth. Nature 525, 206–211. 26331536 10.1038/nature15251 PMC4568559

[21] Blee AM , He Y , Yang Y , Ye Z , Yan Y , Pan Y , Ma T , Dugdale J , Kuehn E , Kohli M *et al*. (2018) TMPRSS2‐ERG controls luminal epithelial lineage and antiandrogen sensitivity in PTEN and TP53‐mutated prostate cancer. Clin Cancer Res 24, 4551–4565. 29844131 10.1158/1078-0432.CCR-18-0653 PMC6139075

[22] Hu S , Wang M , Ji A , Yang J , Gao R , Li X , Sun L , Wang J , Zhang Y and Liu H (2023) Mutant p53 and ELK1 co‐drive FRA‐1 expression to induce metastasis in breast cancer. FEBS Lett 597, 3087–3101. 37971884 10.1002/1873-3468.14774

[23] Pourebrahim R , Zhang Y , Liu B , Gao R , Xiong S , Lin PP , McArthur MJ , Ostrowski MC and Lozano G (2017) Integrative genome analysis of somatic p53 mutant osteosarcomas identifies Ets2‐dependent regulation of small nucleolar RNAs by mutant p53 protein. Genes Dev 31, 1847–1857. 29021240 10.1101/gad.304972.117 PMC5695086

[24] Xiong S , Tu H , Kollareddy M , Pant V , Li Q , Zhang Y , Jackson JG , Suh YA , Elizondo‐Fraire AC , Yang P *et al*. (2014) Pla2g16 phospholipase mediates gain‐of‐function activities of mutant p53. Proc Natl Acad Sci U S A 111, 11145–11150. 25024203 10.1073/pnas.1404139111 PMC4121829

[25] Ding D , Blee AM , Zhang J , Pan Y , Becker NA , Maher LJ 3rd , Jimenez R , Wang L and Huang H (2023) Gain‐of‐function mutant p53 together with ERG proto‐oncogene drive prostate cancer by beta‐catenin activation and pyrimidine synthesis. Nat Commun 14, 4671. 37537199 10.1038/s41467-023-40352-4 PMC10400651

[26] Sizemore GM , Pitarresi JR , Balakrishnan S and Ostrowski MC (2017) The ETS family of oncogenic transcription factors in solid tumours. Nat Rev Cancer 17, 337–351. 28450705 10.1038/nrc.2017.20

[27] Plotnik JP and Hollenhorst PC (2017) Interaction with ZMYND11 mediates opposing roles of Ras‐responsive transcription factors ETS1 and ETS2. Nucleic Acids Res 45, 4452–4462. 28119415 10.1093/nar/gkx039 PMC5416753

[28] Cai C , Hsieh CL , Omwancha J , Zheng Z , Chen SY , Baert JL and Shemshedini L (2007) ETV1 is a novel androgen receptor‐regulated gene that mediates prostate cancer cell invasion. Mol Endocrinol 21, 1835–1846. 17505060 10.1210/me.2006-0480

[29] Adamo P and Ladomery MR (2016) The oncogene ERG: a key factor in prostate cancer. Oncogene 35, 403–414. 25915839 10.1038/onc.2015.109

[30] Baena E , Shao Z , Linn DE , Glass K , Hamblen MJ , Fujiwara Y , Kim J , Nguyen M , Zhang X , Godinho FJ *et al*. (2013) ETV1 directs androgen metabolism and confers aggressive prostate cancer in targeted mice and patients. Genes Dev 27, 683–698. 23512661 10.1101/gad.211011.112 PMC3613614

[31] Ouyang M , Wang H , Ma J , Lu W , Li J , Yao C , Chang G , Bi J , Wang S and Wang W (2015) COP1, the negative regulator of ETV1, influences prognosis in triple‐negative breast cancer. BMC Cancer 15, 132. 25884720 10.1186/s12885-015-1151-y PMC4381371

[32] Mitri ZI , Abuhadra N , Goodyear SM , Hobbs EA , Kaempf A , Thompson AM and Moulder SL (2022) Impact of TP53 mutations in triple negative breast cancer. NPJ Precis Oncol 6, 64. 36085319 10.1038/s41698-022-00303-6 PMC9463132

[33] Selvaraj N , Kedage V and Hollenhorst PC (2015) Comparison of MAPK specificity across the ETS transcription factor family identifies a high‐affinity ERK interaction required for ERG function in prostate cells. Cell Commun Signal 13, 12. 25885538 10.1186/s12964-015-0089-7 PMC4338625

[34] Wolf ER , McAtarsney CP , Bredhold KE , Kline AM and Mayo LD (2018) Mutant and wild‐type p53 form complexes with p73 upon phosphorylation by the kinase JNK. Sci Signal 11, eaao4170. 29615516 10.1126/scisignal.aao4170 PMC6671681

[35] Mabry AR , Gorman J , Delvasto JS , Lavik AR , Layer JH and Mayo LD (2024) Activation of the snail transcription factor induces Mdm2 gene expression. J Biol Chem 300, 107811. 39313097 10.1016/j.jbc.2024.107811 PMC11530585

[36] Greulich BM , Plotnik JP , Jerde TJ and Hollenhorst PC (2021) Toll‐like receptor 4 signaling activates ERG function in prostate cancer and provides a therapeutic target. NAR Cancer 3, zcaa046. 33554122 10.1093/narcan/zcaa046 PMC7848947

[37] Schafer C , Young D , Singh H , Jayakrishnan R , Banerjee S , Song Y , Dobi A , Petrovics G , Srivastava S , Srivastava S *et al*. (2023) Development and characterization of an ETV1 rabbit monoclonal antibody for the immunohistochemical detection of ETV1 expression in cancer tissue specimens. J Immunol Methods 518, 113493. 37196930 10.1016/j.jim.2023.113493 PMC10802095

[38] Adelaiye‐Ogala R , Budka J , Damayanti NP , Arrington J , Ferris M , Hsu CC , Chintala S , Orillion A , Miles KM , Shen L *et al*. (2017) EZH2 modifies Sunitinib resistance in renal cell carcinoma by Kinome reprogramming. Cancer Res 77, 6651–6666. 28978636 10.1158/0008-5472.CAN-17-0899 PMC5712262

[39] Plotnik JP , Budka JA , Ferris MW and Hollenhorst PC (2014) ETS1 is a genome‐wide effector of RAS/ERK signaling in epithelial cells. Nucleic Acids Res 42, 11928–11940. 25294825 10.1093/nar/gku929 PMC4231772

[40] Strittmatter BG , Jerde TJ and Hollenhorst PC (2021) Ras/ERK and PI3K/AKT signaling differentially regulate oncogenic ERG mediated transcription in prostate cells. PLoS Genet 17, e1009708. 34314419 10.1371/journal.pgen.1009708 PMC8345871

[41] Chen X , Zhang T , Su W , Dou Z , Zhao D , Jin X , Lei H , Wang J , Xie X , Cheng B *et al*. (2022) Mutant p53 in cancer: from molecular mechanism to therapeutic modulation. Cell Death Dis 13, 974. 36400749 10.1038/s41419-022-05408-1 PMC9674619

[42] Regan MC , Horanyi PS , Pryor EE Jr , Sarver JL , Cafiso DS and Bushweller JH (2013) Structural and dynamic studies of the transcription factor ERG reveal DNA binding is allosterically autoinhibited. Proc Natl Acad Sci U S A 110, 13374–13379. 23898196 10.1073/pnas.1301726110 PMC3746864

[43] Guedes LB , Almutairi F , Haffner MC , Rajoria G , Liu Z , Klimek S , Zoino R , Yousefi K , Sharma R , De Marzo AM *et al*. (2017) Analytic, Preanalytic, and clinical validation of p53 IHC for detection of TP53 missense mutation in prostate cancer. Clin Cancer Res 23, 4693–4703. 28446506 10.1158/1078-0432.CCR-17-0257 PMC5559307

[44] Tomlins SA , Mehra R , Rhodes DR , Cao X , Wang L , Dhanasekaran SM , Kalyana‐Sundaram S , Wei JT , Rubin MA , Pienta KJ *et al*. (2007) Integrative molecular concept modeling of prostate cancer progression. Nat Genet 39, 41–51. 17173048 10.1038/ng1935

[45] Tomlins SA , Rhodes DR , Perner S , Dhanasekaran SM , Mehra R , Sun XW , Varambally S , Cao X , Tchinda J , Kuefer R *et al*. (2005) Recurrent fusion of TMPRSS2 and ETS transcription factor genes in prostate cancer. Science 310, 644–648. 16254181 10.1126/science.1117679

[46] Chng KR , Chang CW , Tan SK , Yang C , Hong SZ , Sng NY and Cheung E (2012) A transcriptional repressor co‐regulatory network governing androgen response in prostate cancers. EMBO J 31, 2810–2823. 22531786 10.1038/emboj.2012.112 PMC3380210

[47] Liao SY , Rudoy D , Frank SB , Phan LT , Klezovitch O , Kwan J , Coleman I , Haffner MC , Li D , Nelson PS *et al*. (2023) SND1 binds to ERG and promotes tumor growth in genetic mouse models of prostate cancer. Nat Commun 14, 7435. 37973913 10.1038/s41467-023-43245-8 PMC10654515

[48] Volpert M , Furic L , Hu J , O'Connor AE , Rebello RJ , Keerthikumar S , Evans J , Merriner DJ , Pedersen J , Risbridger GP *et al*. (2020) CRISP3 expression drives prostate cancer invasion and progression. Endocr Relat Cancer 27, 415–430. 32357309 10.1530/ERC-20-0092

[49] Burdelski C , Strauss C , Tsourlakis MC , Kluth M , Hube‐Magg C , Melling N , Lebok P , Minner S , Koop C , Graefen M *et al*. (2015) Overexpression of thymidylate synthase (TYMS) is associated with aggressive tumor features and early PSA recurrence in prostate cancer. Oncotarget 6, 8377–8387. 25762627 10.18632/oncotarget.3107 PMC4480759

[50] Cancer Genome Atlas Research Network (2011) Integrated genomic analyses of ovarian carcinoma. Nature 474, 609–615. 21720365 10.1038/nature10166 PMC3163504

[51] Ku SY , Rosario S , Wang Y , Mu P , Seshadri M , Goodrich ZW , Goodrich MM , Labbe DP , Gomez EC , Wang J *et al*. (2017) Rb1 and Trp53 cooperate to suppress prostate cancer lineage plasticity, metastasis, and antiandrogen resistance. Science 355, 78–83. 28059767 10.1126/science.aah4199 PMC5367887

[52] Mu P , Zhang Z , Benelli M , Karthaus WR , Hoover E , Chen CC , Wongvipat J , Ku SY , Gao D , Cao Z *et al*. (2017) SOX2 promotes lineage plasticity and antiandrogen resistance in TP53‐ and RB1‐deficient prostate cancer. Science 355, 84–88. 28059768 10.1126/science.aah4307 PMC5247742

[53] Grupp K , Kohl S , Sirma H , Simon R , Steurer S , Becker A , Adam M , Izbicki J , Sauter G , Minner S *et al*. (2013) Cysteine‐rich secretory protein 3 overexpression is linked to a subset of PTEN‐deleted ERG fusion‐positive prostate cancers with early biochemical recurrence. Mod Pathol 26, 733–742. 23196798 10.1038/modpathol.2012.206

[54] Huang L , Xie Y , Jiang S , Liu K , Ming Z and Shan H (2025) Elucidating the role of pyrimidine metabolism in prostate cancer and its therapeutic implications. Sci Rep 15, 2003. 39814835 10.1038/s41598-025-86052-5 PMC11735813

[55] Ferrie JJ , Karr JP , Graham TGW , Dailey GM , Zhang G , Tjian R and Darzacq X (2024) p300 is an obligate integrator of combinatorial transcription factor inputs. Mol Cell 84, 234–243.e234. 38159566 10.1016/j.molcel.2023.12.004

