# Supplementary material for: Multiple ETS family transcription factors bind mutant p53 via distinct interaction regions
Source: FEBS Lett. 2025 Dec 31;600(8):1248–62. doi: 10.1002/1873-3468.70260 (PMC13113215; doi:10.1002/1873-3468.70260)
Supplement: Supplementary file 1 — Fig. S1. Representative images of purified proteins and binding assay. Fig. S2. ETS truncations interaction with mutant p53. Fig. S3. Analysis of p53 genomic binding. Fig. S4. Patient mutation frequency for all p53 mutations that occur more than once across the Ovarian TCGA Firehose Legacy dataset. Fig. S5. Lung adenocarcinoma dataset. Table S1. shRNA sequences. [file FEB2-600-1248-s001.pdf]

Supplemental Table S1: shRNA Sequences	
shRNA	Sequence
ERG shRNA #1 Forward	ccggAAGGAACTCTCCTGATGAATGctcgagCATTCATCAGGAGAGTTCCTTtttttg
ERG shRNA #1 Reverse	aattcaaaaaAAGGAACTCTCCTGATGAATGctcgagCATTCATCAGGAGAGTTCCTT
ERG shRNA #2 Forward	ccggAAGACTGTCTCATGAGCAAGActcgagTCTTGCTCATGAGACAGTCTTtttttg
ERG shRNA #2 Reverse	aattcaaaaaAAGACTGTCTCATGAGCAAGActcgagTCTTGCTCATGAGACAGTCTT
Control shRNA Forward	CCGGCTTACGCTGAGTACTTCGATTCAAGAGATCGAAGTACTCAGCGTAAGTTTTTTTG
Control shRNA Reverse	AATTCAAAAAAACTTACGCTGAGTACTTCGATCTCTTGAATCGAAGTACTCAGCGTAAG

Supplemental Table S1: shRNA Sequences. Sequences used to generate the shRNA plasmids for either control or ERG knockdown. Control shRNA targets firefly luciferase, which is not expressed in these cells.

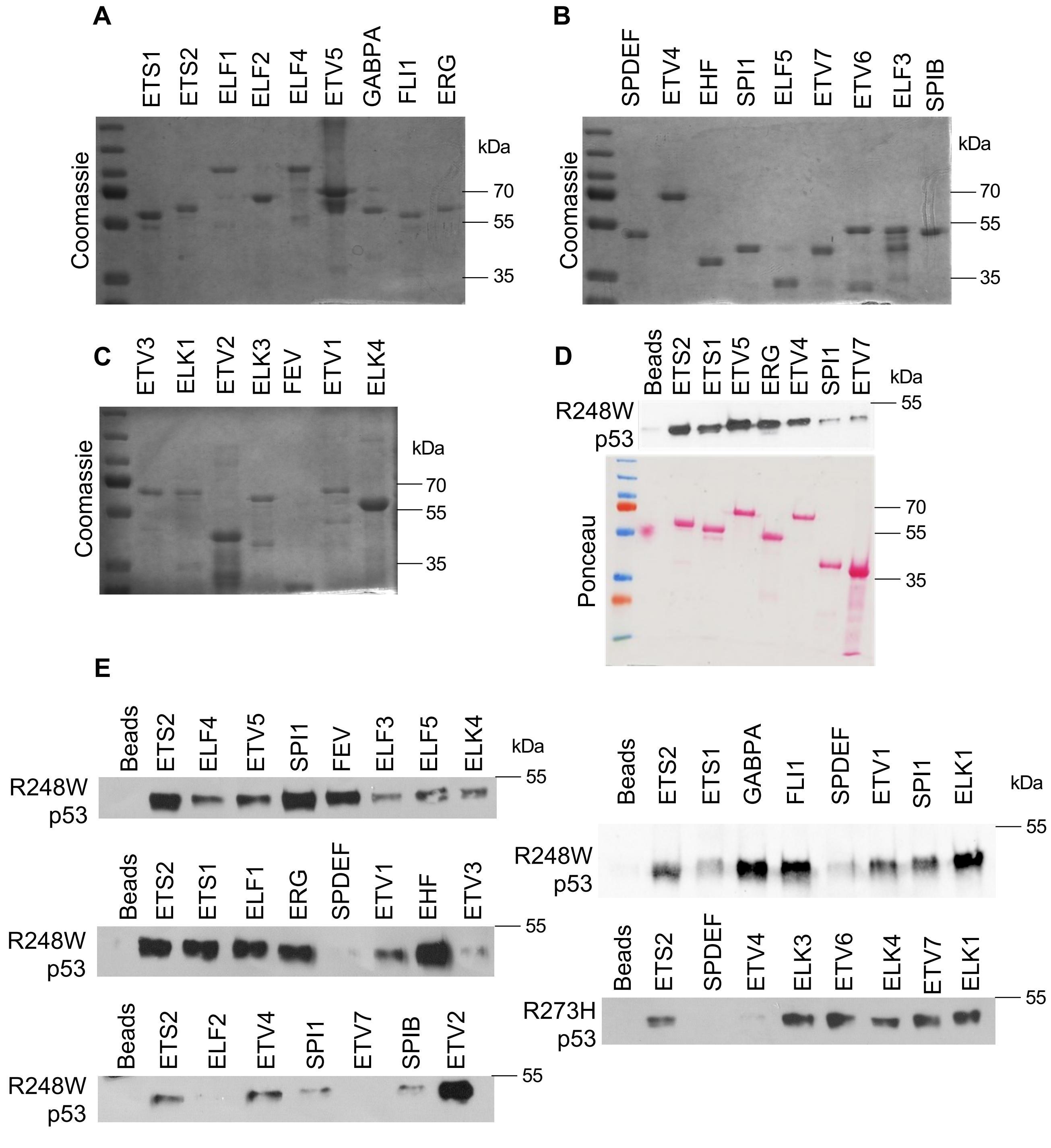

Supplementary Figure S1: Representative Images of Purified Proteins and Binding Assay. A-C) Equal amounts of purified proteins run on an SDS-Page gel and stained with Coomassie stain. Images taken using the EPI setting on a Chemdoc imager. **D)** Representative image of both the ponceau stain for the normalization of the purified ETS proteins and the immunoblot of p53 for the normalization of the amount of bound p53. **E)** Representative images of the immunoblot of p53 following the binding assay with various ETS.

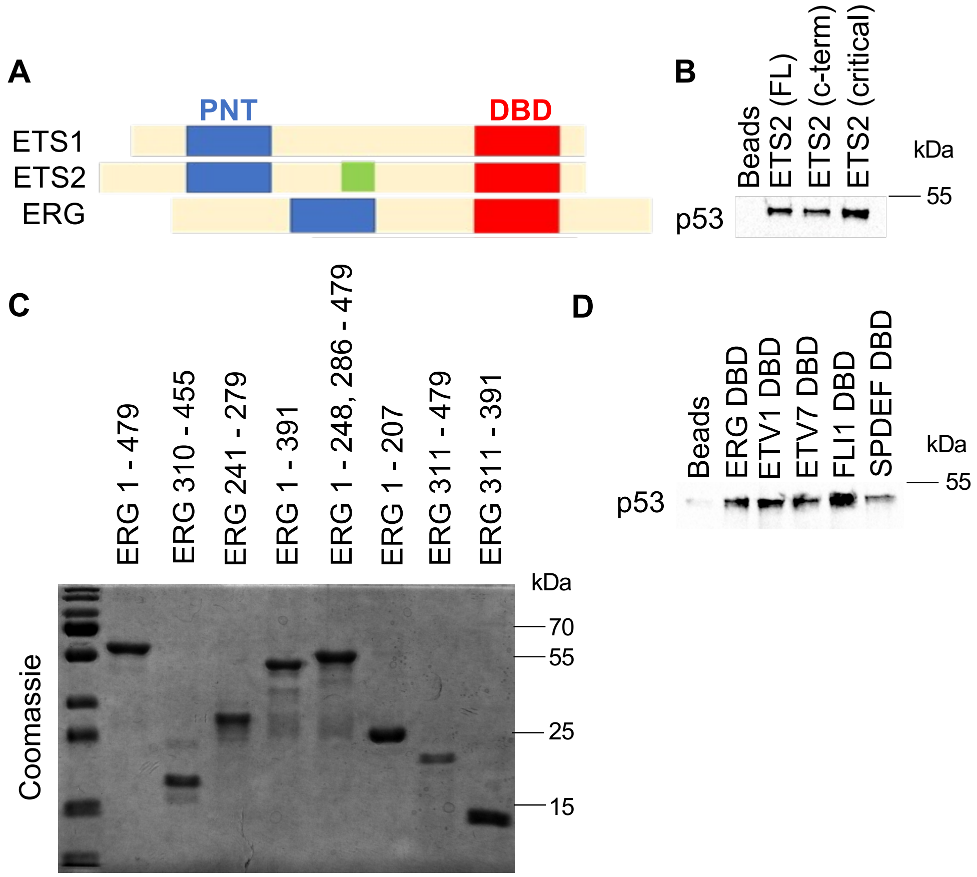

Supplementary Figure S2: ETS truncations interaction with mutant p53. A) Schematic of the identified region of interaction in ETS2 from Do et al. Genes and Dev. 2012 (Green) compared to ETS2's closest homolog (ETS1) and ERG. DNA binding domains and pointed domains shown in red and blue respectively. **B)** Representative image of the immunoblot for p53 for the ETS2 truncations. **C)** Equal loading of ERG truncation loaded into an SDS-Page gel and stained with Coomassie. **D)** Representative image of the immunoblot for p53 for the ETS DNA Binding Domain (DBD) truncations.

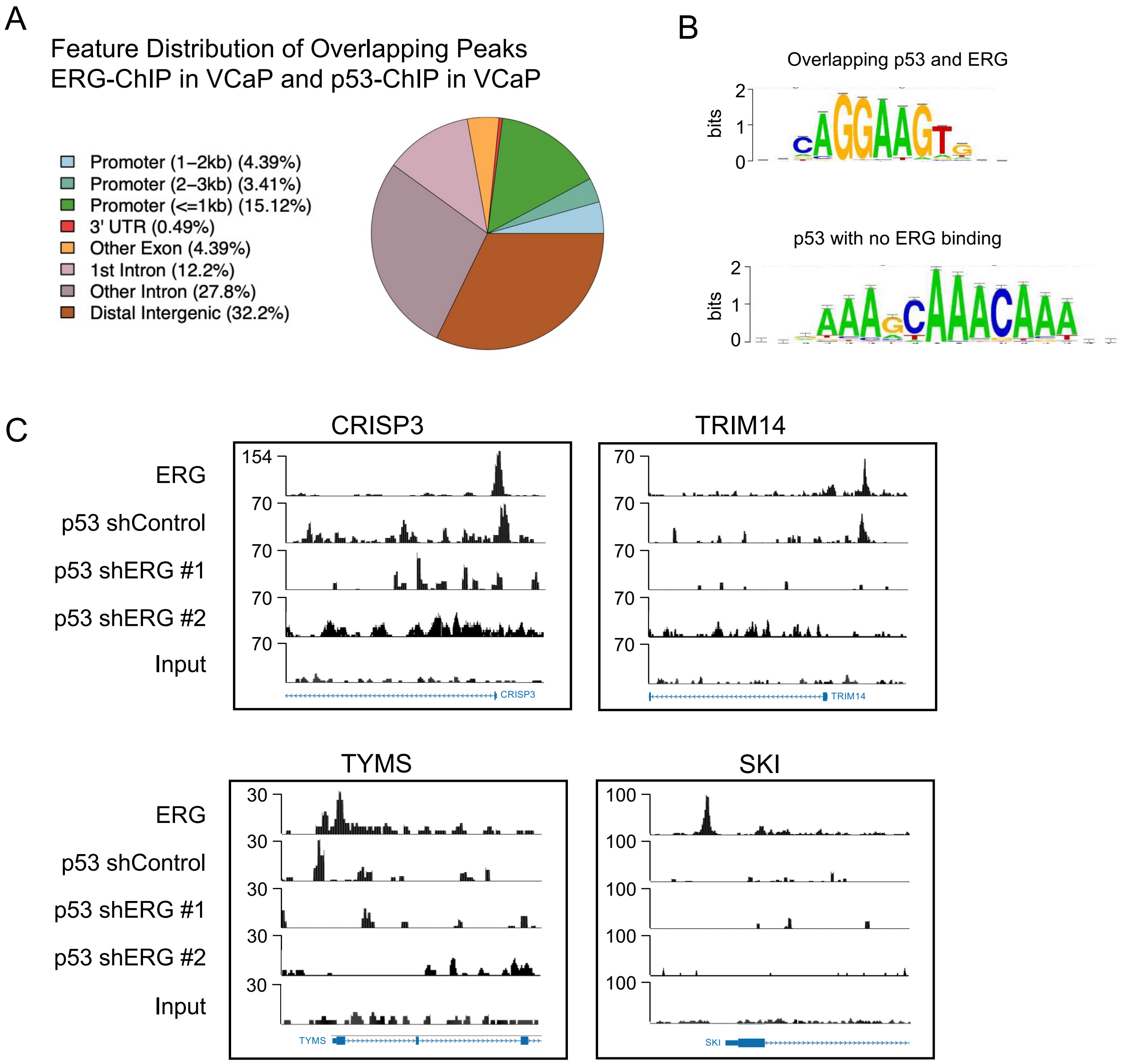

Supplementary Figure S3: Analysis of p53 genomic binding. (A) Genomic distribution of 199 peaks bound by p53 in control, but not ERG shRNA VCaP, and bound by ERG. **(B)** Top most over-represented sequence motif identified in indicated group of bound regions. **(C)** Examples of peaks near four indicated genes. CRISP3, TRIM14, and TYMS have ERG-dependent p53 binding. SKI is bound by ERG but not p53. Relative signal intensity is shown on the y-axis.

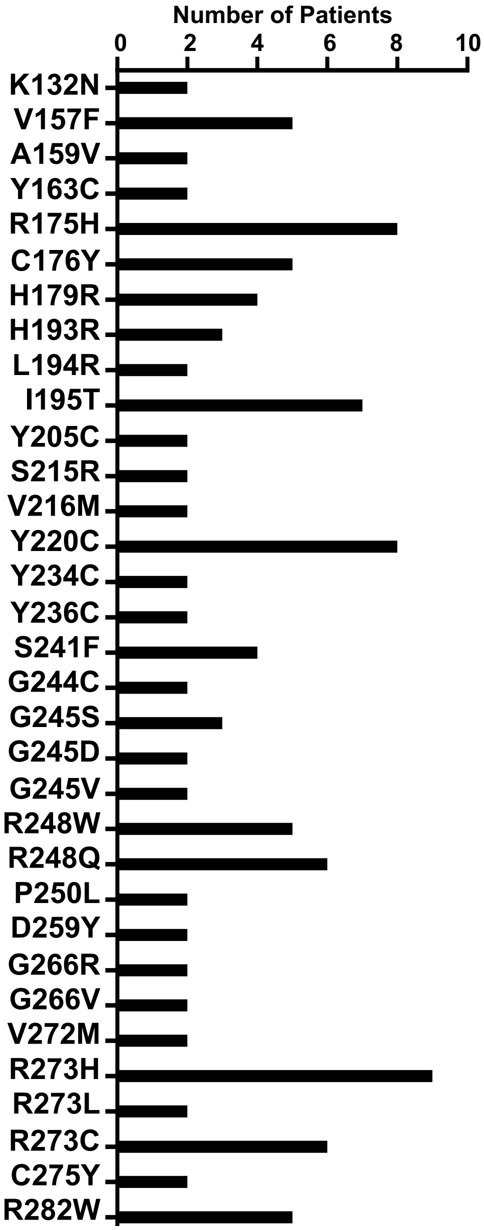

Supplementary Figure S4: Patient mutation frequency for all p53 mutations that occur more than once across the Ovarian TCGA Firehose Legacy dataset.

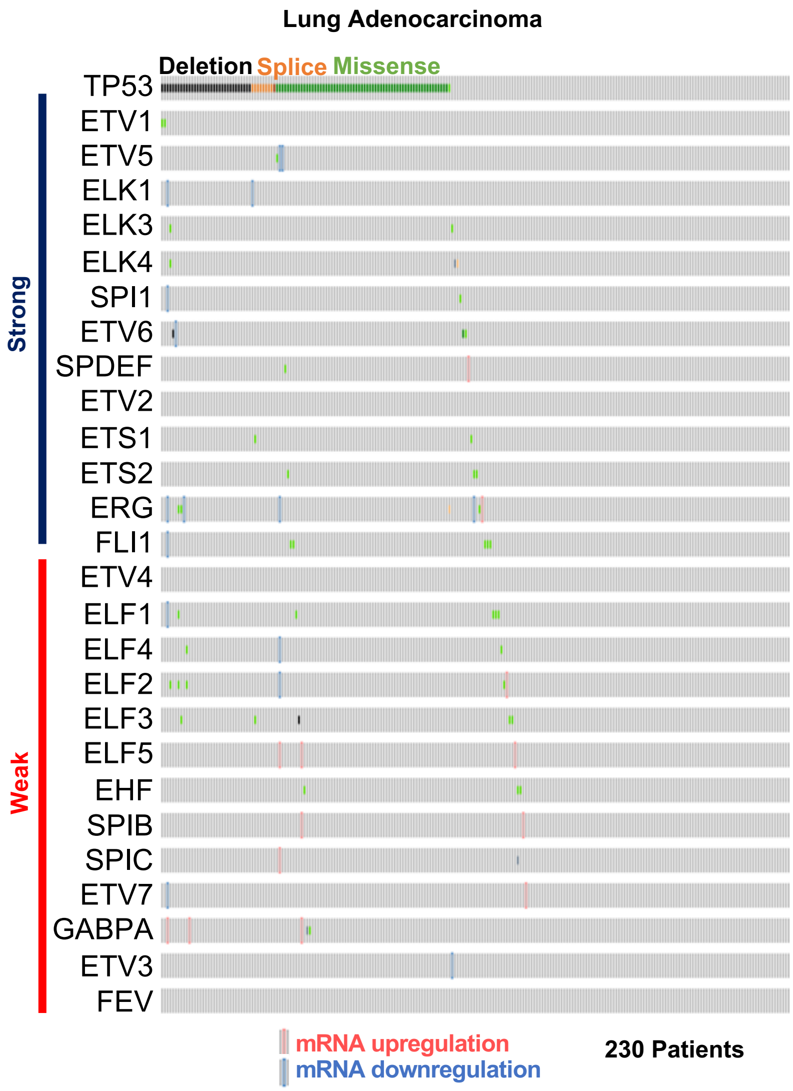

Supplementary Figure S5: Lung Adenocarcinma dataset. Patient data from the TCGA Firehose Legacy dataset for Lung Adenocarcinoma stratified by strong and weak interacting ETS and separated into p53 status of deletion, splice mutant, or missense mutant. mRNA levels are indicated by pink for upregulated and blue for downregulated compared to the average expression level.
